# Human Papillomavirus in Reproductive Health and Pregnancy: Clinical Implications, Outcomes, and a Comprehensive Review of Vaccination

**DOI:** 10.3390/vaccines14020180

**Published:** 2026-02-14

**Authors:** Hasan Volkan Ege, Charlotte Goutallier, Laura Burney Ellis, Houssein El Hajj, Joanna Kacperczyk-Bartnik, Bilal Esat Temiz, Nadja Taumberger, Reda Hemida, Gökçen Ege, Utku Akgör, Zvi Vaknin, Maria Kyrgiou, Murat Gultekin

**Affiliations:** 1 Division of Gynaecological Oncology, Department of Obstetrics and Gynaecology, Faculty of Medicine, Hacettepe University, Ankara 06230, Turkey; drvolkanege@hacettepe.edu.tr (H.V.E.); bilalesattemiz@gmail.com (B.E.T.); utkuakgor@gmail.com (U.A.); 2 Department of Obstetrics and Gynaecology, Mercy Hospital for Women, Victoria 3084, Australia; cgoutallier@mercy.com.au; 3 Department of Digestion, Metabolism and Reproduction-Surgery and Cancer, Institute of Reproductive and Developmental Biology, Faculty of Medicine, Imperial College London, London W12 0NN, UK; laura.ellis@imperial.ac.uk (L.B.E.); m.kyrgiou@imperial.ac.uk (M.K.); 4 Department of Obstetrics & Gynaecology, Imperial College Healthcare NHS Trust, London W2 1NY, UK; 5 Department of Gynecologic Oncology, Gustave Roussy, 94800 Villejuif, France; houssein.el-hajj@gustaveroussy.fr; 6 Department of Gynaecological Oncology, Maria Skłodowska-Curie National Research Institute of Oncology, 02-034 Warsaw, Poland; asiakacperczyk@gmail.com; 7 II Department of Obstetrics and Gynaecology, Medical University of Warsaw, 02-091 Warsaw, Poland; 8 Hospital Barmherzige Brüder Graz, 8020 Graz, Austria; nadja.taumberger@medunigraz.at; 9 Department of Obstetrics and Gynecology, Medical University of Graz, 8010 Graz, Austria; 10 Gynecologic Oncology Unit, Department of Obstetrics and Gynecology, Faculty of Medicine, Mansoura University, Mansoura 35516, Egypt; reda11@mans.edu.eg; 11 Department of Gynecologic Oncology, Ankara Etlik City Hospital, Ankara 06170, Turkey; gokcen.ege@saglik.gov.tr; 12 Department of Obstetrics and Gynecology, The Yitzhak Shamir Medical Center, Zerifin 70300, Israel; vakninz@shamir.gov.il

**Keywords:** adverse pregnancy outcomes, cervical cancer screening, excisional procedure, human papillomavirus, infertility, pregnancy, vaccine

## Abstract

Background/Objectives: Human papillomavirus (HPV) is the most common sexually transmitted virus worldwide and is frequently detected in women of reproductive age. In this population, HPV-related diseases and their management may affect reproductive health and pregnancy outcomes. This narrative review summarizes the current evidence on HPV infection and HPV-related diseases in relation to fertility, pregnancy, and neonatal outcomes, and discusses preventive strategies, with a particular focus on HPV vaccination. Methods: An international, multidisciplinary team of clinicians from the European Society of Gynaecological Oncology (ESGO) Prevention Committee reviewed the literature on HPV, HPV-related diseases, HPV vaccination, and reproductive outcomes, without time restrictions, prioritizing studies judged to meaningfully reflect the available evidence. Results: The most consistent evidence linking HPV-related conditions to adverse pregnancy outcomes relates to the treatment of cervical precancer, particularly excisional procedures, which are associated with an increased risk of preterm birth and mid-trimester pregnancy loss. In contrast, evidence that maternal HPV detection alone causes adverse pregnancy or neonatal outcomes remains limited and inconsistent. Data on HPV infection and subfertility are scarce and heterogeneous. Management of HPV-related lesions during pregnancy remains challenging and requires careful balancing of maternal safety with avoidance of unnecessary interventions. HPV DNA has been detected in neonatal samples, but convincing evidence for clinically relevant vertical transmission is lacking. Available data indicate that inadvertent HPV vaccination shortly before or during pregnancy is not associated with adverse pregnancy outcomes. Conclusions: Current evidence suggests that reproductive risks are more strongly associated with the treatment of HPV-related diseases than with HPV infection itself. Preventive strategies—especially HPV vaccination—remain central to reducing HPV-related disease burden. Although HPV vaccines are not routinely recommended during pregnancy, evidence supports the safety of inadvertent exposure around conception or during gestation, while potential long-term benefits of vaccination regarding reproductive health require further study.

## 1. Introduction

Cervical cancer remains a major global public health problem and is the fourth most common cancer in women worldwide [1]. Persistent infection with high-risk human papillomavirus (HPV) is a necessary cause of virtually all cervical cancers and also contributes to a wider spectrum of anogenital and oropharyngeal diseases.

HPVs are small, non-enveloped, icosahedral DNA-viruses that infect skin and mucosal surfaces in a species-specific manner [2]. More than 200 HPV types have been identified, with at least 50 shown to infect the anogenital tract [2,3]. Twelve types have been officially classified as ‘high-risk’, although approximately 20 HPV types have been associated with cervical cancer based on epidemiological and carcinogenic classification studies [4,5]. HPV 16 and 18 are the most common, and together account for 70% of invasive cervical cancers [6]. HPV types included in the nonavalent vaccine (HPV16/18/31/33/45/52/58) together account for approximately 90% of cervical cancers [7]. HPV prevalence and genotype distribution vary by age, geography, sexual behaviour patterns, and vaccine uptake [8,9].

HPV infection is common, with most individuals acquiring the infection soon after their sexual debut [10,11]. Women aged 20–29 have the highest incidence [12]. Around 90% of cervical HPV infections clear within two years [13]. However, a minority of infections persist and can lead to precancerous lesions (cervical intraepithelial neoplasia, CIN) and, over time, invasive cancer [14]. Clearance of infection and progression of disease depend on factors, including immunity (i.e., HIV, immunosuppressive medications) and genetic factors (i.e., human leukocyte antigen type) [15,16]. The vaginal microbiome has also been shown to be associated with progression or regression in early cervical disease [17,18,19]. Organized HPV-based screening programmes detect precancer and prevent cancer by enabling treatment of clinically significant lesions [20].

Pregnancy introduces specific clinical considerations. It is a state of dynamic immune modulation rather than simple immune deficiency [21]. Both HPV detection and HPV-related lesions are frequently encountered in women of reproductive age. HPV infection and its associated lesions may have adverse effects on pregnancy or reproductive status. The treatment of these lesions can be associated with adverse pregnancy outcomes (APOs). HPV positivity and cervical abnormalities during pregnancy can create anxiety, trigger additional investigations, and pose challenges for clinical management. HPV may raise concerns not only during pregnancy but also in the preconception period. Furthermore, the overlap between the recommended age for HPV vaccination and the age at which pregnancy is desired may influence women’s perceptions of HPV vaccines and lead to lower vaccination rates.

This review summarizes the current evidence on HPV infection and HPV-related disease in relation to fertility, pregnancy, and neonatal outcomes, and discusses the safety and timing of preventive strategies, particularly HPV vaccination, in the context of pregnancy planning and antenatal care.

## 2. Materials and Methods

This review aims to summarize the available studies examining the relationship between HPV infections, HPV-related diseases, and HPV vaccines in the context of fertility, pregnancy, and neonatal outcomes. The review was developed by an international, multidisciplinary group of 12 clinicians from seven countries, all members of the European Society of Gynaecological Oncology (ESGO) Prevention Committee, coordinated by Maria Kyrgiou and Murat Gültekin.

An initial consensus meeting was held to define the scope, key clinical questions, and structure of the review. Relevant literature was identified through searches of PubMed, Web of Science, and the Cochrane Library, without time restrictions. Searches were performed using combinations of keywords covering HPV, pregnancy, fertility, assisted reproductive technologies, cervical precancer, obstetric outcomes, and HPV vaccination.

Priority was given to studies considered most relevant to current clinical practice, including high-quality observational studies, randomized trials, systematic reviews, meta-analyses, and clinical guidelines. Case reports and small case series were not included. In cases where conflicting findings were identified, greater weight was given to high-level evidence, including systematic reviews, meta-analyses, large-scale population-based studies, and methodologically rigorous clinical studies. In topics where the available evidence was heterogeneous, the available literature was summarized in a balanced manner, aiming to reflect the full spectrum of reported findings. As a narrative review, the selection and synthesis of evidence were not conducted according to a predefined systematic protocol, which may introduce a risk of selection bias.

Draft sections were prepared by assigned authors and discussed within the group. Differences in interpretation or emphasis were resolved through consensus. Limitations related to study heterogeneity and evidence quality were considered when interpreting the findings. The final manuscript was reviewed and approved by all authors prior to submission.

## 3. Results

### 3.1. HPV-Related Clinical Conditions

HPV infection is the causative agent of many benign, premalignant, or malignant lesions [22]. Low-risk HPV (lrHPV) types are primarily associated with anogenital warts, whereas high-risk HPV (hrHPV) types, with established oncogenic potential, are responsible for preinvasive and invasive lesions of the anogenital tract, most notably cervical cancer [23,24].

Cutaneous warts, characterized by persistent viral proliferation, are associated with an increased risk of non-melanoma skin cancer [23]. Although anogenital warts generally cause low morbidity, these lesions play a role in the etiology of anal cancer [23,25]. These lesions may also result from non-sexual HPV transmission, especially in children [26].

Persistent hrHPV infection can lead to high-grade precancerous lesions and, if left untreated, to invasive cancer. Cervical cancer is the most common hrHPV-related cancer worldwide [27]. However, substantial decreases in cervical cancer incidence and mortality have been documented primarily in countries with long-standing, organized screening programmes and high population coverage [28]. More recently, HPV-based screening has been shown to provide significantly greater protection against invasive cervical cancer than cytology [29,30].

Oropharyngeal HPV infection, although less frequently addressed, can be associated with both benign and malignant lesions [31]. Lesions such as condyloma acuminatum, squamous papilloma, and verruca vulgaris occur with lrHPV infection of the buccal mucosa. HrHPV infection has been shown to play a role in the development of head and neck cancer, although the relationship appears more significant in higher-income countries [32].

### 3.2. The Impact of HPV-Related Cervical Disease Treatment on Future Pregnancies

Persistent HPV can lead to HPV-related changes, or precancerous changes, in the cervix known as CIN. CIN is divided into three grades corresponding to the depth of changes in the epithelium. CIN2 and CIN3 have the potential to develop into cervical cancer if left untreated; for this reason, surgical treatment with excisional methods should usually be performed for CIN2 or worse lesions diagnosed by cervical biopsy. In 2006, a meta-analysis including 27 studies first highlighted that undergoing surgical treatment of the cervix increased the risk of adverse reproductive outcomes in future pregnancies, including mid-trimester loss, and preterm birth (PTB) [33]. This was corroborated by a Cochrane Review published 10 years later, including 69 studies, which concluded that CIN itself increases the risk of future PTB and midtrimester loss, and that this risk is increased by local surgical treatment [34]. This may be due to a combination of factors, in particular cervical structural changes, but possibly also immunological factors, or even changes in the cervical microbiome [19,35].

**Figure 1:**
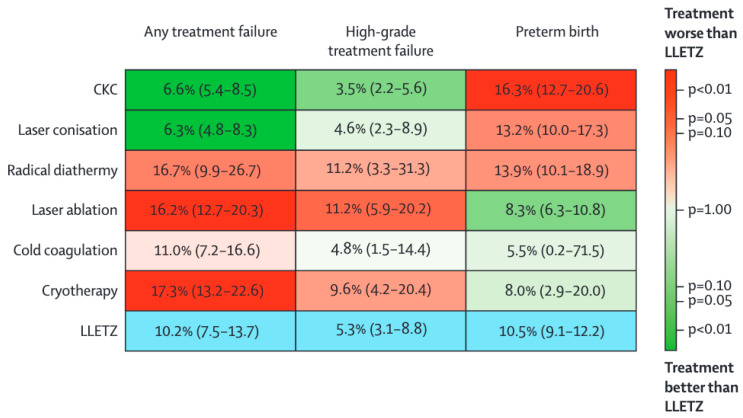
Kilim plot showing the absolute risks of treatment failure and PTB for different options for local treatment of CIN (LLETZ; large loop excision of the transformation zone) [36].

Local surgical treatments include excisional treatments, such as loop excision of the transformation zone and cold knife conization, as well as ablative treatments. A network meta-analysis published in 2022 [36] compared the effectiveness and risk of PTB of local treatments and included 71 studies for treatment failure and 29 studies for PTB. The analysis concluded that whilst more radical excisional treatments reduce the risk of recurrence or invasive disease, they increase the risk of PTB (Figure 1). Cold knife conization is the most radical procedure and carries an absolute risk of treatment failure of 6.6% (95% CI 5.4–8.5) and an absolute risk of PTB of 16.3% (95% CI 12.7–20.6). Ablative treatments likely do not contribute to an increased risk in future pregnancies, but do increase the risk of treatment failure; the absolute risk of PTB with cryotherapy was calculated to be 8.0% (95%CI 2.9–20.0), and the absolute risk of failure was as high as 17.3% (95% CI 13.2–22.6). A loop excision probably carries the most balanced effectiveness and risk of future reproductive morbidity, with a risk of treatment failure of 10.2% (95% CI 7.5–13.7), and risk of PTB of 10.5% (95% CI 9.1–12.2) [36]. Additionally, it has been shown that the size of the local surgical treatment excision proportionally increases this risk while also influencing the future risk of recurrence [37,38]. As the age of childbearing has increased over the last few decades, the age of development of CIN is often around the age at which the first pregnancy occurs [39]. It is, therefore, paramount that both the size and method of the excision or treatment are tailored to the individual patient, their wishes for future fertility, and oncological needs. Recently, the British Society of Colposcopy and Cervical Pathology (BSCCP) and ESGO produced guidelines on active surveillance of CIN2, facilitating evidence-based shared decision-making for patients with CIN2 who are planning a future pregnancy and wish to consider active surveillance [40].

Fertility-sparing treatment for HPV-related cervical cancer less than 2 cm in size is acceptable under ESGO Cervical Cancer Guidelines, which were recently updated in 2023, for those who meet the criteria and wish to retain fertility [41]. Fertility-sparing treatments include conization, simple trachelectomy, radical vaginal trachelectomy, and radical abdominal trachelectomy. Recurrence rates after radical trachelectomy are comparable to those after a radical hysterectomy, although the risk of mid-trimester loss and PTB in subsequent pregnancies is high [42].

Prior to surgical treatment of CIN or cervical cancer, the risks and implications for future pregnancies should be discussed [36,41]. Those who become pregnant post-treatment should be referred to a specialist and be made aware of the signs and symptoms of pre-term labour. In some centres, these patients are screened with tests that predict preterm labour, such as transvaginal cervical length scanning and fetal fibronectin detection [43]. Preventative measures include cervical cerclage and vaginal progesterone, although there is a lack of evidence currently to prove their efficacy. A retrospective study compared the rate of PTB in 725 pregnant women with previous conization to the local and national background rates, and found lower than expected rates for those who received a cerclage [44]. Preliminary evidence suggests that monofilament may be preferable to braided sutures [45].

While HPV in the vulva and vagina also leads to precancerous changes known as vulvar or vaginal intraepithelial neoplasia (VIN or VaIN), these diseases are significantly less common than CIN and additionally tend to affect older women; therefore, they rarely present in women of childbearing age. While treatment of these lesions prior to pregnancy is not thought to have any risks in future pregnancies, it is recommended that treatment be postponed until after delivery for lesions identified during pregnancy [46].

### 3.3. HPV Infection in Pregnant Women

The high prevalence of HPV infection among pregnant women means that its potential impact on maternal and fetal health is crucial to understand. The rates of HPV infection in this demographic are understood to be slightly higher than in non-pregnant women and can be attributed to hormonal or immunological changes during pregnancy [47,48]. These changes during pregnancy may affect the natural progression of HPV by influencing the persistence and clearance of the infection. In 2022, Ardekani et al. researched the prevalence of cervical–vaginal HPV in a meta-analysis involving 45,077 pregnant women from 39 countries. Their analysis reported a pooled global HPV prevalence of 30.38% (95% CI, 26.88–33.99%), with marked regional variation, ranging from 16.91% in the Eastern Mediterranean to 46.46% in Africa (*p* < 0.001). Furthermore, the study highlighted the association between a country’s income level and HPV prevalence, noting rates of 67.73% in low-income, 34.27% in lower-middle-income, 36.01% in upper-middle-income, and 23.85% in high-income countries [49]. These rates were significantly higher than a previous meta-analysis by Liu et al. in 2014, which estimated a global HPV prevalence in pregnant women at 16·82% (95% CI, 16.21–17.47%) [50]. Younger pregnant women (under 20–25 years old) are especially vulnerable to HPV, with strains 16 and 18 being the most prevalent in all groups, according to both studies. The HPV types detected in pregnant women may vary across different geographic regions [47]. These findings mainly reflect unvaccinated or partially vaccinated populations. Genotype distribution among younger women may differ in highly vaccinated cohorts depending on vaccine uptake and type [9,51]. Additionally, some population-based studies have demonstrated that increasing HPV vaccination coverage is associated with substantial changes in the distribution of circulating HPV genotypes. Marked reductions in vaccine-targeted types, particularly HPV-16 and HPV-18, have been observed not only among vaccinated women but also among unvaccinated individuals within the same population [52,53]. This phenomenon is largely attributed to herd effects resulting from reduced viral transmission. Consequently, in theory, the genotype profile detected in pregnant women may also progressively shift away from the vaccine HPV types in highly vaccinated settings, potentially altering the relative predominance of specific HPV types over time. Most studies focused on detecting HPV DNA in the uterine cervix of pregnant women; HPV DNA has also been detected in pregnancy-related tissues, a finding that has raised questions regarding possible mother-to-child transmission, which is discussed in detail in later sections [49,54,55].

Anatomical, cardiovascular, metabolic, hormonal, and immunological changes are among the many adaptive changes that pregnancy brings about in order to support and tolerate the semi-allogeneic fetus. This immunological tolerance is characterized by alterations in immune cell populations, including a reduction in natural killer cells and a decrease in helper T-cell type 1-mediated immunity [55,56]. In addition, some studies have identified steroid hormone receptor–binding elements within the HPV-16 transcriptional promoter, raising the possibility that the hormonal milieu of pregnancy may influence HPV transcriptional activity [50]. Moreover, hrHPV types feature glucocorticoid response elements in their viral upstream regulatory regions, suggesting that these viruses could increase gene transcription and expression in response to the elevated steroid hormone levels characteristic of pregnancy [55]. Several studies have shown that trophoblast cells are susceptible to several HPV strains, including HPV 16, 18, 11, and 31, supporting biological interaction between HPV and placental tissues without necessarily implying clinically relevant vertical transmission [57,58,59].

Antimicrobial peptides, such as α-defensins, play a crucial role in protecting the host against HPV by preventing HPV virion release, L2 protein cleavage, facilitating lysosomal degradation of HPV capsids, and reducing local pro-inflammatory cytokine production [60,61]. However, due to the regulatory control of estradiol, α-defensin production is decreased during pregnancy, particularly in the third trimester [62]. Furthermore, during pregnancy, macrophages, which are key players in immune surveillance, increase in number. Interestingly, HPV has been detected in placental macrophages, suggesting that the virus can influence and be influenced by the unique immune environment during pregnancy. The cytotoxic activity of decidual natural killer cells activated against virus-infected cells decreases against HPV-infected trophoblasts. This change may demonstrate HPV’s potential to avoid immune surveillance in the placenta [63].

### 3.4. Screening, Diagnosis, and Treatment During Pregnancy

#### 3.4.1. Screening

Pregnancy and the inevitable healthcare interactions associated with it may represent an opportunity to offer cervical cancer screening to women who are underscreened or not up to date with screening programmes. Pap smear sampling during pregnancy has been shown to be procedurally safe and does not adversely affect pregnancy outcomes [64]. However, hormonal and immune changes can complicate cytology interpretation. Pap smears undertaken during pregnancy may show benign changes (i.e., Arias–Stella reaction, decidual cells), making diagnosis challenging [65]. However, the move towards primary HPV testing reduces reliance on cytology testing [66]. Cervical cancer screening during pregnancy should continue to follow national and organized screening recommendations [67]. In most countries, the incidence of positive screening tests and the management of screen-positive women is generally similar in pregnancy and outside of pregnancy [68].

#### 3.4.2. Diagnosis and Treatment

In pregnancy, the primary goal of colposcopy is to exclude invasive disease. When colposcopic evaluation is limited in early pregnancy due to migration of the transformation zone, re-evaluation can be made between 6 and 12 weeks later, potentially allowing for a sufficient evaluation of the transformation zone by the early second trimester [69]. Performing colposcopy in pregnant women may be challenging due to factors such as vaginal expansion, the risk of supine hypotension, increased risk of gastroesophageal reflux, and, in the later stages of pregnancy, cervical effacement and dilation [70]. Examinations should therefore be performed only by experienced colposcopists who are familiar with both pregnancy-related and neoplastic cervical changes [64].

Pregnant women without evidence of high-grade disease on cytology, colposcopy, or biopsy can be re-evaluated at least 4 weeks postpartum. However, for those diagnosed with high-grade lesions, regular monitoring with colposcopy and age-based testing (cytology/HPV) every 12–24 weeks is recommended [64,71]. The length of the follow-up interval can be personalized, based on factors such as the gestation, clinician expertise, and risk of loss to follow-up [72]. Cervical biopsies can be safely performed during pregnancy without a significant increase in the risk of excessive bleeding. If bleeding occurs, it can be managed with Monsel’s solution or sutures [73]. Treatment of preinvasive lesions is not recommended during pregnancy [74]. Conization is recommended when microinvasive disease or in situ adenocarcinoma (stage IA or microscopic IB, no clinically visible lesion) is detected on biopsy to exclude invasive cancer. This procedure can be performed using either the loop electrosurgical excisional procedure or the cold-knife conization method [75,76]. However, extensive excision of the epithelium and underlying stroma within the endocervical canal is not recommended due to the risk of hemorrhage, miscarriage, premature rupture of membranes, preterm labour/delivery, and infection [77]. Endocervical sampling is also not recommended during pregnancy.

#### 3.4.3. New Screening Methods

Due to the challenges in cytological evaluation of Pap smears during pregnancy, research has been conducted to investigate whether p16/Ki-67 dual staining could aid as a triage test in this setting [78,79,80]. Trutnovsky et al. reported that the sensitivity and specificity of p16/Ki-67 dual-stained cytology for CIN2+ in women with abnormal Pap cytology were 100% and 66.7%, respectively [81]. However, Ciavattini et al. found excessive p16/Ki-67 staining in pregnant women irrespective of CIN grade. It was concluded that the staining did not provide an accurate representation of the lesion’s severity, often exaggerating the degree of the lesion beyond its actual extent [82]. Current evidence is therefore insufficient to recommend routine p16/Ki-67 dual staining for cervical cancer screening during pregnancy.

Studies conducted on non-pregnant women have demonstrated high detection rates of FAM19A4/miR124-2 methylation in cervical cancer and advanced CIN2/3 lesions (95–100%) [83,84]. Additionally, the absence of FAM19A4/miR124-2 methylation during pregnancy was associated with the absence of any CIN2/3 lesions over a 14-year follow-up period [85]. IHampl et al. demonstrated that cases with a negative FAM19A4/miR124-2 methylation test during pregnancy could safely continue monitoring until the postpartum period without the risk of disease progression, thereby avoiding unnecessary treatment during pregnancy [86]. However, because evidence remains limited, routine use of FAM19A4/miR124-2 methylation testing in pregnant women cannot yet be recommended.

Self-sampling for hrHPV has been shown to have similar accuracy to clinician-taken samples in the general population and may represent an acceptable alternative for women who decline screening. Current data are insufficient to recommend their use in pregnancy. Urine-based testing presents another option, which, although less accurate than vaginal samples, may allow for the extension of screening to underscreened populations. More evidence on the accuracy of urine-based HPV testing is needed [87,88].

### 3.5. The Relationship Between HPV Infection and Fertility

Sexually transmitted infections are thought to be responsible for more than a third of cases of female infertility, often by inducing pelvic inflammation and tubal obstruction [89]. HPV is, however, not a sexually transmitted infection capable of causing pelvic inflammatory disease, and is far less likely biologically to have any impact on female fertility. While some small studies have suggested a potential negative association between HPV infection and male infertility, its effects on female infertility are even less well understood [90,91,92,93]. Several cross-sectional studies have shown varying rates of HPV detection in infertile women compared to their fertile counterparts, with HPV being detected 3–5 times more frequently in infertile individuals [94,95]. Additionally, women with subfertility are nearly twice as likely to exhibit HPV-related abnormal cytology or high-grade cervical lesions [96]. However, these associations may reflect confounding (e.g., sexual behaviour, co-infections), surveillance bias, and reverse causation rather than a direct effect of HPV on fecundity [97,98]. In a meta-analysis of >15,000 women, the overall association between HPV and female infertility was not statistically significant, although high-risk HPV (hrHPV) infection showed a significant association with infertility, highlighting the need for cautious interpretation and better-controlled prospective studies [98].

In contrast, the evidence linking HPV to male reproductive parameters is more consistent. HPV DNA can be detected in semen, and meta-analyses suggest that semen HPV positivity is associated with impaired semen quality (particularly reduced motility and abnormal morphology) and may contribute to male-factor infertility [90,91,92,99]. Proposed mechanisms include direct binding of HPV to spermatozoa with potential viral transfer to the oocyte/embryo, increased sperm DNA fragmentation, and local inflammatory/immune effects (including anti-sperm antibodies) [90,97].

Accordingly, multiple studies have assessed assisted reproductive technology (ART) outcomes in HPV-positive couples. Recent meta-analyses suggest that HPV detected in semen is associated with reduced clinical pregnancy rates and/or an increased risk of early pregnancy loss after IVF/ICSI, although effect estimates vary and certainty is limited by small study sizes, heterogeneous HPV assays/sampling sites, variable ART protocols, and incomplete adjustment for confounders [93,97,100]. More recent prospective data also suggest that semen HPV infection may negatively influence semen parameters and ART outcomes, but residual confounding and limited event numbers remain important limitations [101].

Numerous small studies have highlighted a reduction in fertility outcomes in HPV-infected couples undergoing Assisted Reproductive Technology (ART). This has been theoretically attributed to the HPV having a possible negative impact on various stages of human embryo development. One in vitro study demonstrated that introducing the HPV type 16 genome into trophoblasts increased apoptosis, potentially leading to placental dysfunction, miscarriage, and premature rupture of membranes. Further robust in vitro and clinical studies are necessary before any true potential adverse effect of hrHPV on fertility and early pregnancy development in patients undergoing ART can be implied [97].

Several meta-analyses have further explored the association between HPV infection and outcomes of assisted reproductive technologies, including in vitro fertilization (IVF) and intracytoplasmic sperm injection (ICSI). A meta-analysis including eight studies reported that HPV infection detected in semen was associated with male fertility abnormalities, suggesting a potential negative impact of HPV on male reproductive function [92]. However, the available evidence regarding the effects of HPV infection on pregnancy rates and miscarriage following ART remains limited and inconclusive [93]. Given limitations in study design and measurement, the relationship between HPV and subfertility is still uncertain and requires confirmation in higher-quality prospective studies. At present, routine HPV testing in semen or in the female genital tract solely for infertility or ART evaluation cannot be recommended and should be regarded as investigational, pending further high-quality prospective evidence, outside established cervical cancer screening programmes. While some data suggest an association between high-risk HPV infection and female infertility, evidence regarding low-risk HPV types remains even more sparse, and this too requires confirmation in well-designed prospective studies [97,98].

Furthermore, a study examining 190 women undergoing treatment for CIN2 or CIN3 found that IVF/ICSI outcomes were unaffected. The authors concluded that women undergoing local surgical treatment of the cervix can proceed with ART without delay post-treatment [102].

Despite the lack of evidence to support the link between HPV and fertility-related outcomes, timely HPV vaccination before sexual debut could be beneficial to negate any possible negative effects of HPV, including those not fully established. HPV vaccines themselves are not associated with an increase in the risk of premature ovarian failure [103]. The potential benefit of HPV vaccination needs to be supported by large-scale epidemiological studies.

### 3.6. HPV and Pregnancy Outcomes

HPV infection, associated lesions, or their treatment have the potential to affect pregnancy outcomes.

#### 3.6.1. Miscarriage

Evidence regarding HPV infection and the risk of miscarriage is weak and has shown mixed results. A study including 143 pregnant women reported an increased prevalence of HPV in spontaneously aborted products of conception [104]. A systematic review including 42 studies concluded that HPV infection was associated with a higher risk of miscarriage [105]. However, other studies have reported that HPV infection is not associated with miscarriage [106]. A systematic review and meta-analysis published in 2020 found no association between cervical HPV infection and the risk of miscarriage [107]. In fact, lower HPV rates were reported among patients with recurrent miscarriage [108]. Substantial heterogeneity in control groups, study design, sampling timing, and HPV detection methods may underlie the inconsistent findings, and again, this may represent association as opposed to causation.

#### 3.6.2. Preterm Labour

It is well-established that treatment of HPV-related cervical lesions increases the risk of preterm labour, especially with excisional treatment [38]. This may be explained by the increased risk of premature rupture of membranes [58]. However, HPV infection itself in early pregnancy was not associated with increased risk of PTB without preterm premature rupture of membranes (PPROM) [109]. Moreover, a data linkage study in Scotland found no evidence that HPV infection or low-grade HPV-associated cervical disease was associated with PTB [110].

#### 3.6.3. Preterm Premature Rupture of Membranes

In a large retrospective study including 400,583 singleton deliveries, HPV infection was significantly associated with PPROM [111]. This association has been corroborated by a number of other studies [58,112]. This may simply be an association with other vaginal infections or, indeed, vaginal microbiome changes. However, a smaller study of 20 patients reported that HPV did not increase the risk of PPROM [113].

#### 3.6.4. Fetal Growth Restriction (FGR)

Recent studies and systematic reviews report a correlation between the presence of maternal HPV and FGR [58,114,115,116]. This is possibly due to high-risk HPV-related lymphohistiocystic villitis (HPV-LHV), HPV infection in the trophoblastic tissue, or Hofbauer cells of the placenta [58,114]. However, these associations do not establish causality and are heavily influenced by confounding obstetric and maternal factors.

### 3.7. HPV Transmission

Vertical transmission of HPV from mother to fetus could theoretically occur in three ways: peri-conceptually, prenatally, and perinatally. Importantly, the detection of HPV DNA in pregnancy-related tissues or in neonates cannot necessarily indicate true infection and may reflect transient exposure or contamination rather than clinically meaningful vertical transmission. Concordance between maternal and infant HPV types has been used to speculate that vertical transmission could occur [117]; however, horizontal transmission from relatives or peers after birth may also explain these findings [118] and may be more likely in the context of our current understanding. Although several theoretical pathways for vertical transmission have been proposed, the currently available literature provides heterogeneous evidence regarding vertical transmission and does not allow definitive conclusions regarding its frequency or clinical relevance. The majority of studies in this context have not differentiated between viral deposition and true infection.

#### 3.7.1. Peri-Conceptual

Peri-conception transmission has been theorized as potentially occurring through infected sperm or, less commonly, through infected oocytes [119]. In a 2017 systematic review, HPV was detected in 11.4% of semen samples, and in 20.4% of semen samples for men referred for investigation of infertility [120]. Commonly, studies have focused on whether sperm from HPV-infected males is associated with adverse pregnancy outcomes, rather than directly assessing transmission to the fetus [120,121]. HPV infection has been shown to be associated with reduced sperm motility, DNA fragmentation, and anti-sperm antibodies, although these studies are small and there are many potential confounding factors [122,123,124]. Sperm washing and HPV vaccination in infected men has been shown to reduce seminal HPV load, and one small retrospective study reported improved pregnancy outcomes following vaccination. However, this finding may reflect spontaneous viral clearance or differences in baseline characteristics rather than a causal effect of vaccination [123,125]. More research is needed to determine the presence of HPV DNA in oocytes and its effects on pregnancy, if any exist [119]. Overall, current evidence is insufficient to demonstrate clear vertical transmission of HPV during the periconceptional period.

#### 3.7.2. Prenatal

HPV DNA has been detected in pregnancy-related tissues, including the placenta, amniotic fluid, and chorionic tissue, leading to speculation about possible prenatal transmission. A recent meta-analysis reported a prevalence of 30.38% for HPV in cervicovaginal samples, 17.81% in placental tissue, and 2.26% in amniotic fluid [49].

In the prospective HERITAGE cohort study, including 1050 pregnant women, HPV was detected in 40.2% of vaginal samples and in 10.7% of placentas. However, HPV detection beneath the amniotic membrane (on the fetal side) was substantially lower (3.9%). HPV DNA was identified in 7.2% of neonates at birth or within the first three months, most commonly in conjunctival samples, but was not detected beyond six months of age. These findings suggest that neonatal HPV detection is most often transient, rather than indicating true infection [126]. The discrepancy in HPV detection rates between placental compartments may in fact reflect contamination during delivery or differences in sampling techniques. Supporting this, studies using transabdominal placental sampling have reported much lower HPV positivity rates (2/35 cases) [59]. In a prospective cohort study conducted with 153 pregnant women, HPV positivity in the placenta was detected at 3.3% [127]. However, the detection of HPV DNA on the fetal side of the amniotic membrane in the HERITAGE study does suggest possible placental involvement [126]. Overall, the detection of HPV in the placenta varied significantly between studies, particularly with sampling techniques. In conclusion, HPV-positive placentas are more likely to reflect sample contamination than true infection, either when the placenta comes into contact with the vaginal canal or due to inaccurate sampling methods.

Evidence regarding HPV DNA in cord blood is inconsistent. In a study involving 153 pregnant women, 36.6% of whom were HPV-positive, HPV was not detected in cord blood [128]. Several studies have reported the absence of HPV detection in cord blood [129]. In the study involving 315 pregnancies, HPV positivity in cord blood was detected at 3.5%. The presence of abnormal cytology increased the risk of HPV positivity in cord blood [130]. This variability may also be attributable to differences in sampling techniques or contamination, and the clinical significance of these findings remains unclear.

Several studies have reported the detection of HPV DNA in intrauterine tissues, suggesting that in utero exposure to HPV DNA may occur. However, most neonatal detections appear to be transient and clear spontaneously, suggesting that persistent or clinically meaningful prenatal infection is uncommon and remains unproven. Further well-designed studies distinguishing viral deposition from true infection are required to clarify the role and clinical relevance of prenatal HPV exposure.

#### 3.7.3. Perinatal

Perinatal HPV transmission may occur through contact with infected maternal genital tract cells during vaginal delivery. One systematic review and meta-analysis suggested higher rates of HPV detection in neonates who were born by vaginal delivery in comparison to those born by cesarean section (28.2% vs. 14.9%; RR 0.52, 95% CI 0.34–0.78) [131]. However, a recent meta-analysis found no significant association between mode of delivery and HPV transmission (pooled RR 0.91, 95% CI 0.23–3.67) [132].

Studies evaluating viral persistence suggest that HPV detected in neonates after birth is usually transient. In one study reporting a vertical transmission rate of 20.8%, HPV was no longer detectable in neonatal samples obtained two months later, consistent with findings from the HERITAGE study [129]. These data indicate that perinatally detected HPV most likely reflects temporary viral deposition or transient colonization, although the possibility of true infection in a small subset of cases cannot be completely excluded.

The clinical significance of perinatal HPV detection remains uncertain. There is no clear evidence that cesarean section prevents persistent HPV infection or HPV-related disease in neonates; therefore, cesarean delivery should not be routinely recommended for the prevention of HPV transmission. Cesarean section may be indicated in rare circumstances, for example, in women with anogenital warts causing pelvic outlet obstruction, or if vaginal birth is thought to carry a high chance of excessive bleeding due to friable tissues [133].

Current evidence does not suggest that breastfeeding influences HPV persistence or clearance. Postpartum immune reconstitution may aid clearance; however, based on the currently available evidence, HPV remains unaffected by breastfeeding [134].

Taken together, the available data are too weak to confirm the theoretical possibility of vertical exposure. Existing studies are limited by methodological heterogeneity and an inability to consistently distinguish viral deposition from true infection. The clinical significance remains even more uncertain, and further research is needed to clarify whether perinatal HPV exposure could result in clinically meaningful infection or long-term consequences for the infant. Overall, while vertical transmission of HPV is biologically plausible and transient neonatal detection has been documented, current evidence suggests that clinically meaningful and persistent neonatal infection is rare.

### 3.8. HPV Infection in the Newborn and in Early Childhood

HPV may lead to skin and mucosal lesions, Juvenile Recurrent Respiratory Papillomatosis, Retinoblastoma, and Conjunctival Papilloma [49]. Skin lesions, known as verruca vulgaris, may occur anywhere on the body and are common in school-aged children [135]. Mucosal lesions may be benign or malignant. The most common benign lesions are oral squamous papillomas [136]. Malignant mucosal lesions are rare in the pediatric population [137].

Juvenile recurrent respiratory papillomatosis, secondary to perinatal transmission of mainly HPV-6 and HPV-11 [138], involves the recurrent growth of papillomas in the upper respiratory tract [139]. Clinical presentation ranges from mild symptoms with subsequent remission to lung involvement with significant morbidity [139]. The relationship between HPV infection and retinoblastoma is still debated. HPV has been detected in intraocular samples of patients with retinoblastoma; however, reported detection rates vary, with some studies finding HPV prevalence below that of the general population. Conjunctival papilloma is a benign tumour of the conjunctiva and represents 1–10% of lesions in the conjunctiva in children and adolescents [140].

While historically some studies [129,141,142,143] report ongoing HPV detection in infants from birth, these positive results are at short time frames of usually less than six months postnatally. One study suggested persistence at 3 years, and even at 6 years post birth, albeit in very small numbers [144]. A study in Finland reported a 21% seroconversion rate for neonates born to HPV-negative mothers by 3 years of age, suggesting HPV acquisition during early childhood rather than vertical transmission [145]. It is likely that the persistence of HPV, when acquired in the perinatal period, is rare, as there is growing evidence [126,129,141,142,143] suggesting clearance of HPV within the first years of life when initially detected in the perinatal period [145]. It should be acknowledged that HPV positivity detected at an early stage of life may reflect transient deposition of viral DNA or surface contamination rather than a true infection. Further data is needed to confirm whether the virus is truly cleared or if it remains undetected and may reactivate later in life.

### 3.9. HPV Vaccines and Pregnancy

HPV vaccines were first approved by the U.S. Food and Drug Administration in 2006 and have since been administered to millions of women worldwide [146]. The vaccine plays an important role in preventing both persistent HPV infections and HPV-related lesions [147]. HPV vaccination has been demonstrated to reduce the risk of anogenital precancerous lesions and cancers [24,148]. Recent population-based studies have shown that HPV vaccination is associated with a substantial reduction in the incidence of invasive cervical cancer in vaccinated cohorts [149,150]. Many institutions and organizations, including the World Health Organization (WHO), recommend HPV vaccination prior to sexual debut [151,152]. Although the primary target group is 9–14 years of age, routine vaccination is recommended up to age 26 [153]. This recommended vaccination window overlaps with the early reproductive years, during which many women may be planning pregnancies. As a result, a significant number of reproductive-age women are exposed to the HPV vaccine [154]. This has raised concerns about the potential effects of HPV vaccination on pregnancy. These concerns have focused primarily on the potential adverse effects of vaccination during preconception or pregnancy on the fetus or pregnancy outcomes. A review of vaccines that can be administered during pregnancy—such as COVID-19, influenza, tetanus, and hepatitis B—found that the main reasons for hesitancy among pregnant women were fear of side effects and lack of confidence in vaccine safety [155]. Similarly, studies in non-pregnant populations report that concerns about potential side effects and inadequate information are the most frequently cited reasons for HPV vaccine hesitancy [156]. Fears about potential effects on pregnancy may cause some women to avoid or postpone the HPV vaccine, reducing its effectiveness in preventing cervical cancer and precancerous lesions [146].

The WHO and vaccine manufacturers do not currently recommend HPV vaccination during pregnancy due to a lack of sufficient safety data [151,157,158]. Although routine pregnancy testing prior to vaccination is not a requirement [151,153]. Many women have inadvertently received the HPV vaccine during pregnancy, most commonly during the first trimester [159]. Theoretical concerns have been raised regarding this, including the vaccine’s potential to increase the risk of APOs, such as miscarriage, PTB, or congenital anomalies. Studies evaluating the effects of HPV vaccination on pregnancy outcomes are summarized in Table 1.

A study published in 2017, including between 2315 and 8870 pregnancies, found no significant association between HPV vaccine exposure during pregnancy and an increased risk of major birth defects, miscarriage, PTB, low birth weight, or stillbirth [160]. Similarly, a study involving 895 pregnant women reported that exposure to the quadrivalent HPV vaccine (4vHPV) during pregnancy did not increase the risk of miscarriage [161]. Another large cohort study involving 7487 vaccinated pregnant women also found no increased risk of miscarriage, stillbirth, or infant death within the first year of life among those who received the 4vHPV vaccine, including vaccinations administered within four weeks of conception [162].

The absence of a significant association between HPV vaccination during pregnancy and an increased risk of miscarriage is also supported by many other studies [163,164,165,166,167]. In a cohort of 935 pregnant women, HPV vaccination during pregnancy did not significantly affect the rate of congenital anomalies, mean birth weight, or gestational age at delivery, and the rate of miscarriage was comparable to that of the general population [168]. Similarly, a study evaluating chorioamnionitis, pregnancy-induced hypertension, gestational diabetes, PTB, or SGA birth found that HPV vaccination administered on or after the menstrual period (LMP) was not associated with an increased risk of APOs [169]. Consistent with these findings, several studies have shown that HPV vaccination during pregnancy is not associated with an increased incidence of APOs [164,165,166,167]. During pregnancy, exposure to the nonavalent HPV vaccine (9vHPV) has not been associated with spontaneous abortion, major birth defects, or PTB [170]. Exposure to HPV vaccination during pregnancy was not associated with an increased risk of miscarriage or other APOs, according to a systematic review and meta-analysis, including 11 studies, published in 2024 [146]. Nevertheless, despite these reassuring findings, many women of reproductive age continue to express concern about the potential effects of HPV vaccination on future pregnancies.

Another concern among women of reproductive age is the potential adverse impact of HPV vaccination on pregnancies in the more distant future.

Kharbanda et al. compared women vaccinated 16–22 weeks before pregnancy with those vaccinated within 42 days of the last menstrual period (LMP) and found similar rates of miscarriage after 4vHPV vaccination (10.4% vs. 11.2%) [161]. In a long-term safety study of the bivalent HPV vaccine (2vHPV), the relative risk (RR) of miscarriage for vaccinations administered within 60 days of pregnancy was 1.60 (95% CI: 0.99–2.61), which is comparable to rates in the general population [168]. Although the authors could not completely exclude a slightly increased risk of miscarriage in pregnancies conceived within three months of vaccination, the overall results were encouraging.

In a placebo-controlled trial of 4vHPV, no significant differences were observed between the vaccine and placebo groups in miscarriages (18.2% vs. 19.5%), late fetal loss (<1% in both), or fetal abnormalities (2.0% vs. 1.5%) [171]. Analyses of women who became pregnant within 30 days of vaccination additionally found no higher risk of miscarriages (18.2% vs. 21.0%). There was no increased risk of miscarriages, even in pregnancies that occurred within 90 days after vaccination, according to a study assessing the administration of 2vHPV at any point prior to pregnancy [172]. A small but statistically significant increase in second-trimester (13–20 weeks) miscarriages was noted in one subgroup analysis; however, the authors attributed this finding to potential analytical artefacts from multiple sensitivity analyses.

Further comparisons of distal (4–18 months before conception) and peripregnancy (from 2 weeks before to after LMP) HPV vaccination showed no significant differences in the rates of PTB (7.6% vs. 7.4%), SGA (11.1% vs. 11.8%), or major birth defects (1.8% vs. 1.8%) [169]. Another study comparing distal (4–18 months before conception) and peripregnancy (30 days before to 45 days after conception) HPV vaccination found no significant differences in the rates of miscarriages (9.0% vs. 11.6%), birth defects (6.0% vs. 5.9%), stillbirth (0.6% vs. 0.8%), or SGA births (6.5% vs. 6.0%) [165].

In an evaluation of 9vHPV vaccination outcomes, no increased risks were found for miscarriages (6.0% vs. 4.4%; RR 0.72), PTB (8.1% vs. 5.7%; RR 0.72), SGA birth (6.4% vs. 7.0%; RR 1.10), or major structural birth defects (1.0% vs. 1.1%; RR 1.03) when comparing distal (16–22 weeks before LMP) and prepregnancy (within 42 days before LMP) vaccination [170]. These findings are consistent with earlier 4vHPV trials, suggesting that pre-pregnancy administration of 9vHPV does not increase the risk of APOs.

In a study comparing pregnancy outcomes in women vaccinated with 4vHPV versus placebo, miscarriages and congenital anomaly rates were similar between the groups, and no serious fetal or infant adverse effects were attributed to vaccination [173]. A recent systematic review and meta-analysis reported that exposure to 2vHPV within 45 days of LMP and exposure to 9vHPV between 90 days before and 45 days after LMP were both significantly associated with an increased risk of miscarriages (RR 1.59, 95% CI 1.04–2.45; RR 2.04, 95% CI 1.28–3.24, respectively) [146]. Nevertheless, none of the three vaccines was associated with a significantly elevated risk of other APOs. Overall, HPV vaccination, whether given prior to or during pregnancy, was not linked to an increased risk of miscarriages, birth defects, or PTB, according to another systematic review and meta-analysis published in 2023 [179].

Given the potential adverse effects of HPV infection and HPV-related cervical disease on fertility and pregnancy outcomes, an important question is whether HPV vaccination could indirectly influence these outcomes. However, only a limited number of studies have explored this association, and available evidence remains largely observational. An evaluation of the national HPV vaccination programme in Australia found that for every 20% increase in vaccination coverage in the maternal cohort, there was a corresponding 1% decrease in the rates of PTB and low birth weight, and a 2% decrease in the rate of SGA infants [174]. The positive effect on PTB rates may be due to the decrease in precancerous lesion treatments with HPV vaccines. However, the study did not adjust for the effects of risk factors for APOs (such as smoking and body mass index).

HPV vaccination may lower the risk of PTB (OR 0.61, 95% CI 0.34–1.09), with a particularly significant decrease in early PTB (*p* = 0.04), according to a population-based study from Finland [175]. Another study found that women vaccinated before the age of 17 had a lower risk of PTB (aOR 0.87, 95% CI 0.75–1.00), but those vaccinated at the age of 17 or older showed no such protective effect [176]. Another Finnish population-based cohort study demonstrated a lower incidence of PTB among vaccinated primiparas compared with unvaccinated women (4.1% vs. 5.2%; OR 0.79, 95% CI 0.59–1.04). The authors proposed that a decreased need for cervical excisional treatments after HPV vaccination may serve as an indirect cause of this protective effect [177]. These population-based studies have reported lower rates of PTB among vaccinated women; however, these findings should be interpreted cautiously due to potential residual confounding and lack of adjustment for established risk factors. The need for more research in this area is highlighted by the fact that some studies have found no appreciable improvement in pregnancy outcomes among vaccinated populations [178].

The literature indicates that HPV vaccination is safe during the preconception period. Although no significant APOs have been reported with HPV vaccination during pregnancy, stronger evidence is still required. However, if pregnancy is discovered after vaccination, patients should be informed that HPV vaccines have not been associated with any APOs. Further evidence is needed to determine whether vaccination can truly improve APOs associated with HPV infection itself, HPV-related lesions, or lesion treatment.

Immune responses to vaccination are influenced by sex hormones, which undergo substantial changes during pregnancy and may theoretically affect vaccine immunogenicity [180]. Nevertheless, due to the exclusion of pregnant individuals from HPV vaccine immunogenicity trials, evidence directly comparing immune responses in pregnant and non-pregnant populations remains unavailable. In contrast, immunogenicity data from postpartum women are reassuring, as a non-inferiority trial demonstrated robust antibody responses following a two-dose HPV vaccination series administered in the postpartum period [181].

If a woman becomes pregnant after initiating the HPV vaccination schedule, the remaining doses should be postponed until after the pregnancy. Postpartum follow-up visits may provide an ideal opportunity to initiate or complete the HPV vaccination schedule [182].

Collectively, evidence on inadvertent HPV vaccination during pregnancy is derived predominantly from large population-based cohort studies, post hoc analyses of randomized trials, and recent systematic reviews and meta-analyses. According to available data, receiving an HPV vaccination before conception is generally safe and does not increase the risk of APOs. Even though the literature that is currently available has not shown that inadvertent vaccination during pregnancy causes serious adverse effects, large-scale, well-designed, planned studies would be beneficial to support these findings with robust data. Reports suggesting potential benefits of population-level vaccination on pregnancy outcomes remain observational and require confirmation. Irrespective of this, earlier vaccination remains beneficial for the woman, and the preconception period may represent an opportunity to discuss HPV vaccination with unvaccinated women who are within the recommended age group. For women who are inadvertently exposed to HPV vaccination during pregnancy, reassurance is appropriate, as available evidence does not indicate any substantial risk to the pregnancy or developing fetus. In such cases, remaining vaccine doses should be postponed and administered after delivery. Postpartum visits provide an important opportunity to initiate or complete HPV vaccination, thereby extending protection against HPV-related diseases without compromising maternal or fetal safety. Breastfeeding is not a contraindication to HPV vaccination and should not delay postpartum immunization.

## 4. Conclusions

HPV is common among women of reproductive age. Evidence consistently shows that adverse pregnancy outcomes are associated with excisional treatment of cervical precancer. There is currently no conclusive evidence to suggest that HPV detection alone correlates with an increased risk of poor pregnancy outcomes. In contrast, evidence linking maternal HPV infection itself to adverse pregnancy or neonatal outcomes remains limited and inconsistent, and well-designed, adequately powered studies are still needed.

Antenatal care may offer an opportunity to reach underscreened women, but cervical screening in pregnancy should follow national programme recommendations and screening intervals to avoid unnecessary testing and follow-up in women at low risk. When cervical cancer is suspected, assessment should be performed promptly by experienced clinicians, ideally in specialized settings.

Timely HPV vaccination reduces HPV-related disease and should remain a primary focus in healthcare systems worldwide. Although HPV vaccines have been in use for many years and have demonstrated a safety profile in non-pregnant women, they are not currently recommended for use during pregnancy. Importantly, there is no evidence suggesting an association between HPV vaccination administered either immediately before or during pregnancy and APOs. Therefore, pregnant women who are inadvertently vaccinated should be provided with appropriate counselling to address potential concerns, and any remaining doses should be completed in the postpartum period. Vaccination prior to sexual debut remains the optimum strategy for disease prevention, safety, and cost-effectiveness.

## Figures and Tables

**Table 1:** Summary of studies evaluating pregnancy outcomes following HPV vaccination.

Author	Type of Vaccine	Results
Scheller [160]	4vHPV	Major birth defects (OR 1.19, 95% CI 0.90–1.58), miscarriage (HR 0.71, 95% CI 0.45–1.14), PTB (OR 1.15, 95% CI 0.93–1.42), low birth weight (OR 1.10, 95% CI 0.85–1.43), small for gestational age (SGA) (OR 0.86, 95% CI 0.72–1.02), stillbirth (HR 2.43, 95% CI 0.45–13.21); no increased risk observed.
Kharbanda [161]	4vHPV	The risk of miscarriage was not increased among women who received the vaccine during pregnancy (aHR 1.10, 95% CI 0.81–1.51) or peripregnancy (aHR 1.07, 95% CI 0.81–1.41).
Faber [162]	4vHPV	No significantly increased rate of miscarriage. No association between vaccination during pregnancy and stillbirth (aOR 0.96, 95% CI 0.57–1.61), or infant mortality (aHR 0.94, 95% CI 0.53–1.67).
Goss [163]	4vHPV	The rate of miscarriage was 6.7% (95% CI 5.5–8.2), and the prevalence of major birth defects was 2.4% (95% CI 1.7–3.3), both comparable to background rates in the general population.
Moreira [164]	9vHPV	The proportion of pregnancies with APOs (miscarriage or congenital anomaly) was consistent with those reported in the general population.
Baril [165]	2vHPV	No evidence of an increased risk of miscarriage (aHR 1.30, 95% CI 0.79–2.12) or other adverse pregnancy outcomes was observed in vaccinated women.
Bukowinski [166]	4vHPV	No increased risk was observed between exposure to vaccine during pregnancy and preeclampsia/eclampsia (aHR 1.00, 95% CI 0.80–1.24), preterm labour (aHR 0.92, 95% CI 0.76–1.13), miscarriage (aHR 1.05, 95% CI 0.94–1.18), PTB (aRR 0.87, 95% CI 0.71–1.07), birth defects (aRR 0.67, 95% CI 0.47–0.96), growth problems in utero (aRR 1.21, 95% CI 0.96–1.52), or growth problems in infancy (aRR 0.98, 95% CI 0.76–1.28).
Sy [167]	4vHPV	No safety signal was identified for congenital anomalies or miscarriage following 4vHPV exposure during pregnancy, and the incidence of congenital anomalies was consistent with published background rates.
Angelo [168]	2vHPV	The HPV and control groups were similar in terms of mean birth weight, gestational age at delivery, and rates of congenital anomalies. The miscarriage rate was 15.3% in the vaccine group and 11.1% in the control group (RR 1.37, 95% CI 0.94–2.01).
Lipkind [169]	4vHPV	Vaccination during pregnancy or the periconceptional period was not associated with adverse obstetric or neonatal outcomes; preterm birth occurred in 7.9% vs. 7.6% of pregnancies (aRR 0.97, 95% CI 0.72–1.30), and major structural birth defects in 2.0% vs. 1.8% (aPR 1.0, 95% CI 0.52–1.90).
Kharbanda [170]	9vHPV	Vaccination during pregnancy was not associated with miscarriage (HR 1.12, 95% CI 0.66–1.93), preterm birth (RR 0.73, 95% CI 0.44–1.20), or SGA birth (RR 1.31, 95% CI 0.78–2.20), with similar findings for peripregnancy exposure.
Garland [171]	4vHPV	No significant differences were observed between vaccinated and placebo groups in the proportions of pregnancies resulting in live birth, fetal loss, miscarriage, or congenital anomalies (*p* = 0.20).
Panagiotou [172]	2vHPV	No evidence that vaccination affects the risk of miscarriage in pregnancies conceived within 90 days after vaccination (RR 1.02, 95% CI 0.78–1.34). No increase in abortion risk was found with vaccination at any time, except for miscarriage occurring between weeks 13 and 20 (RR 1.35, *p* = 0.017) after multiple subgroup analyses.
Chen [173]	4vHPV	Ectopic pregnancy (0.4% vs. 1.0%), miscarriage (10.9% vs. 9.0%), late fetal death (1.1% vs. 0.3%), and congenital anomalies (1.3% vs. 0.5%) were similar between the vaccine and placebo groups.
Yuill [174]	4vHPV	HPV vaccination coverage of 60–80% was associated with a relative reduction of 3.2% (95% CI 1.1–5.3%) in PTB and 9.8% (95% CI 8.2–11.4%) in SGA infants.
Kalliala [175]	2vHPV	In the first pregnancy, the PTB rate was 3.2% among vaccinated women versus 5.1% among non-vaccinated women (OR 0.61, 95% CI 0.34–1.09), with early PTB significantly lower in the vaccinated group (*p* = 0.04).
McClymont [176]		Lower likelihood of preterm birth when vaccinated before age 17 (aOR 0.87, 95% CI 0.75–1.00); no association if vaccinated ≥17 years (aOR 1.04, 95% CI 0.98–1.10).
Koivisto [177]	2vHPV	A lower incidence of PTB among vaccinated primiparas compared with unvaccinated women (4.1% vs. 5.2%; OR 0.79, 95% CI 0.59–1.04).
Xu [178]		No significant reduction in PTB, low birth weight, or pre-labour preterm rupture of membranes was observed in the vaccinated group.

Abbreviations: APO, adverse pregnancy outcome; PTB, preterm birth; SGA, small for gestational age; OR, odds ratio; aOR, adjusted odds ratio; RR, relative risk; HR, hazard ratio; CI, confidence interval.

## Data Availability

Not applicable.

## Abbreviations

APOs	Adverse pregnancy outcomes
CIN	Cervical intraepithelial neoplasia
LLETZ	Large loop excision of the transformation zone
VIN	Vulvar intraepithelial neoplasia
VaIN	Vaginal intraepithelial neoplasia
ART	Assisted Reproductive Technology
IVF	In vitro fertilization
ICSI	Intracytoplasmic sperm injection
PPROM	Preterm premature rupture of membranes
PTB	Preterm birth
FGR	Fetal growth restriction
LMP	Last menstrual period
SGA	Small for gestational age
